# Response to comments on our article (Yin YL et al., Parasit Vectors, 10.1186/s13071-021-04739-w) by Yuqing Wang and colleagues

**DOI:** 10.1186/s13071-021-04996-9

**Published:** 2021-09-21

**Authors:** Yan-Ling Yin, Xin Yang, Guang-Hui Zhao

**Affiliations:** Department of Parasitology, College of Veterinary Medicine, Northwest A&F University, Yangling, China

**Keywords:** *Cryptosporidium parvum*, Statistical procedure, Microarray analysis, ciRS-7

## Abstract

This letter responds to comments on our article (Yin YL et al., Parasit Vectors, 10.1186/s13071-021-04739-w) by Yuqing Wang and colleagues, who wrote a letter entitled “Microarray analysis of circular RNAs in HCT-8 cells infected with *Cryptosporidium parvum*” and discussed statistical procedures for microarray analysis during *C. parvum* infection. To further confirm our data, in this letter, a common R package for analyses of differentially expressed genes, namely DESeq2, with Benjamini-Hochberg correction, was used to analyze our microarray data and identified 26 significantly differentially expressed circRNAs using adjusted *P* value < 0.05 and | Log_2_ (fold change [FC]) | ≥ 1.0, including our circRNA ciRS-7 of interest. Therefore, the protocol for selecting circRNAs of interest for further study in our article is acceptable and did not affect the subsequent scientific findings in our article.

**Supplementary Information:**

The online version contains supplementary material available at 10.1186/s13071-021-04996-9.

## To the Editor

Cryptosporidiosis, caused by *Cryptosporidium* spp., is an important zoonotic parasitic disease, seriously threatening the health of humans and many animals [1, 2]. Considering that the severity of cryptosporidiosis is closely associated with host status, especially immunity, understanding the host response to infection is critical to effectively develop well-directed control strategies against cryptosporidiosis [3].

Microarray analysis is a sensitive tool to accurately investigate differentially expressed circRNAs during many disease processes [4–8]. It is based on hybridization and fluorescence detection and uses specific probes targeting the back-spliced junction of each circRNA. CapitalBio Technology Human CircRNA Array, which was used in our study, also has its own analyzing software, GeneSpring, and GeneSpring supports normalization processing and analysis of differential circRNAs of original data from microarray analysis. Therefore, we used this software to process our microarray data. Due to the generally mild impact of *Cryptosporidium* infection on host cell gene transcription compared to other pathogens [9–12], to obtain as many differentially expressed genes as possible, we chose fold changes > 2 combined with *P* ≤ 0.05 as the threshold to preliminarily screen differentially expressed circRNAs in the microarray analysis in our article [3]. The criterion of *P* <0.05 or *P* ≤ 0.05 was also used by recent studies [13–16]. From the subsequent verification of the experimental results, circRNAs of interest obtained using the threshold of *P* ≤ 0.05 are stable and accurate. Certainly, the unadjusted *P*-value will introduce false positives during multiple comparisons, as stated by Wang et al. [17], in their Letter to the Editor published commenting on our article. To reduce this possibility, expression of the circRNA of interest, ciRS-7, was further verified by using real-time PCR in multiple repetitions in our article. This protocol is also a standard method to select genes of interest for subsequent experiments [15, 16, 18, 19].

Nowadays, several software packages and models have been used to define significantly differentially expressed (DE) genes during multiple comparisons. Three R packages, namely limma, DESeq2 and edge R, are commonly used to analyze DE genes for microarray and RNA-seq data. Of these, both limma and DESeq2 are quite reliable and are not much different for multiple comparisons of these data, with > 90% of genes detected overlapping between the two methods [20]. It should be noted that limma can find the accurate DE genes better but obtains fewer significant DE genes because of its rigorous screening criteria, while DESeq2 is more suitable when more possible candidate DE genes are expected. However, both methods are preliminary screening tools that will inevitably introduce false-positive/negative results to a certain extent. Therefore, the convincing expression of candidate DE genes should be further confirmed by other more precise methods with multiple repetitions, such as the real-time PCR used in our article. Since *Cryptosporidium* infection has a mild impact on host cell gene transcription, here we further analyzed our microarray data by using DESeq2 for multiple comparisons using Benjamini-Hochberg correction and identified a total of 26 significantly DE circRNAs (Additional file 1: Table S1) using the standard of adjusted *P* value < 0.05 and | Log_2_ (fold change [FC]) | ≥ 1.0, including our circRNA of interest, ciRS-7 (*P* = 0.030272316 and Log_2_ FC = 2.497972579).

Collectively, the protocol for selecting circRNAs of interest for further study in our article is acceptable and did not affect the subsequent scientific findings. We welcome further discussions of our article.

## Supplementary Information

**Additional file 1**: **Table S1**. Significantly differentially expressed circRNAs between *C. parvum*-infected and -non-infected HCT-8 cells.

## Data Availability

All datasets generated for this study are included in the article and its additional file.

## References

[1] Zhang G, Zhang Y, Niu Z, Wang C, Xie F, Li J (2020). *Cryptosporidium parvum* upregulates miR-942-5p expression in HCT-8 cells via TLR2/TLR4-NF-κB signaling. Parasit Vectors.

[2] Carter BL, Chalmers RM, Davies AP (2020). Health sequelae of human cryptosporidiosis in industrialised countries: a systematic review. Parasit Vectors.

[3] Yin YL, Liu TL, Yao Q, Wang YX, Wu XM, Wang XT (2021). Circular RNA ciRS-7 affects the propagation of *Cryptosporidium parvum* in HCT-8 cells by sponging miR-1270 to activate the NF-κB signaling pathway. Parasit Vectors.

[4] Luo S, Deng M, Xie Z, Li X, Huang G, Zhou Z (2021). Circulating circular RNAs profiles associated with type 1 diabetes. Diabetes Metab Res Rev.

[5] Kalifu B, Maitiseyiti A, Ge X, Chen X, Meng Y (2021). Expression profile of circular RNAs in cystic echinococcosis pericystic tissue. J Clin Lab Anal.

[6] Li Y, Zhang Y, Zhang S, Huang D, Li B, Liang G (2020). circRNA circARNT2 suppressed the sensitivity of hepatocellular carcinoma cells to cisplatin by targeting the miR-155-5p/PDK1 Axis. Mol Ther Nucleic Acids.

[7] Yi F, Xin L, Feng L (2021). Potential mechanism of circRNA_000585 in cholangiocarcinoma. J Int Med Res.

[8] Peng D, Hou ZL, Zhang HX, Zhang S, Zhang SM, Lin RY (2021). Microarray expression profile and analysis of circular RNA regulatory network in pulmonary thromboembolism. Int J Gen Med.

[9] Deng M, Lancto CA, Abrahamsen MS (2004). *Cryptosporidium parvum* regulation of human epithelial cell gene expression. Int J Parasitol.

[10] Yang YL, Serrano MG, Sheoran AS, Manque PA, Buck GA, Widmer G (2009). Over-expression and localization of a host protein on the membrane of *Cryptosporidium parvum* infected epithelial cells. Mol Biochem Parasitol.

[11] Zhou R, Hu G, Liu J, Gong AY, Drescher KM, Chen XM (2009). NF-kappaB p65-dependent transactivation of miRNA genes following *Cryptosporidium parvum* infection stimulates epithelial cell immune responses. PLoS Pathog.

[12] Zhao GH, Gong AY, Wang Y, Zhang XT, Li M, Mathy NW (2018). Nuclear delivery of parasite Cdg2_FLc_0220 RNA transcript to epithelial cells during *Cryptosporidium parvum* infection modulates host gene transcription. Vet Parasitol.

[13] Zhi F, Ding Y, Wang R, Yang Y, Luo K, Hua F (2021). Exosomal hsa_circ_0006859 is a potential biomarker for postmenopausal osteoporosis and enhances adipogenic versus osteogenic differentiation in human bone marrow mesenchymal stem cells by sponging miR-431-5p. Stem Cell Res Ther.

[14] Gao YJ, Zhang RJ, Liu Q, Sun SG, Qi MY, Wang Y (2021). Functional predication of differentially expressed circRNAs/lncRNAs in the prefrontal cortex of Nrf2-knockout mice. Aging (Albany NY).

[15] Li L, Wei H, Zhang H, Xu F, Che G (2020). Circ_100565 promotes proliferation, migration and invasion in non-small cell lung cancer through upregulating HMGA2 via sponging miR-506-3p. Cancer Cell Int.

[16] Li Y, Shi P, Zheng T, Ying Z, Jiang D (2020). Circular RNA hsa_circ_0131242 promotes triple-negative breast cancer progression by sponging hsa-miR-2682. Onco Targets Ther.

[17] Wang Y, Zhao H, Zhang Y, Yan L (2021). Microarray analysis of circular RNAs in HCT-8 cells infected with *Cryptosporidium parvum*. Parasit Vectors.

[18] Xiang Q, Kang L, Wang J, Liao Z, Song Y, Zhao K (2020). CircRNA-CIDN mitigated compression loading-induced damage in human nucleus pulposus cells via miR-34a-5p/SIRT1 axis. EBioMedicine.

[19] Shi Y, Fang N, Li Y, Guo Z, Jiang W, He Y (2020). Circular RNA LPAR3 sponges microRNA-198 to facilitate esophageal cancer migration, invasion, and metastasis. Cancer Sci.

[20] Tong Y (2021). The comparison of limma and DESeq2 in gene analysis. E3S Web Conf..

